# Tree functional composition, functional diversity, and aboveground biomass show dissimilar trajectories in a tropical secondary forest restored through assisted natural regeneration

**DOI:** 10.1002/ece3.9870

**Published:** 2023-03-12

**Authors:** Enock Ssekuubwa, Wouter van Goor, Martijn Snoep, Kars Riemer, Fredrick Wanyama, Daniel Waiswa, Fred Yikii, Mnason Tweheyo

**Affiliations:** ^1^ Department of Forestry, Biodiversity and Tourism Makerere University Kampala Uganda; ^2^ Face the Future Wageningen The Netherlands; ^3^ Uganda Wildlife Authority Kampala Uganda; ^4^ Department of Geography, Geo‐informatics and Climatic Sciences Makerere University Kampala Uganda; ^5^ Department of Environmental Management Makerere University Kampala Uganda

**Keywords:** agricultural abandonment, ecosystem functioning, passive restoration, restoration success, trajectory analysis, Uganda

## Abstract

The growing trend of agricultural abandonment requires an understanding of the development of secondary forests on old fields in the context of restoration. However, few studies examine the regeneration trajectories of functional composition and functional diversity in afrotropical secondary forests. We tested how functional composition, diversity, and aboveground biomass (AGB) change with age and determined restoration success for a secondary forest restored through assisted natural regeneration in Uganda. We assessed the influence of distance to forests on regeneration. We sampled trees in 63 plots (2000 m^2^ each) in the secondary forest (16–22‐year old) and five plots in an old‐growth forest in 2011, 2014 and 2017. We computed functional composition (community‐weighted means—CWM) and diversity using categorical (habitat type, dispersal mode, fruit size, and successional group) and continuous traits (wood density and maximum height) of the species and calculated AGB. The secondary forest showed dissimilar trajectories of functional composition, diversity, and AGB. After 16–22 years, the secondary forest had not yet reached equivalent values of most attributes of functional composition, diversity and AGB in the old‐growth forest. The distance to forests had a negative effect on CWM of forest‐dependent species, nonpioneer light demanders, and functional divergence and a positive effect on CWM of pioneer species. We show that assisted natural regeneration can enhance the functional composition, functional diversity, and AGB of degraded forests and that continued monitoring is needed to attain full recovery. In planning passive restoration, sites closer to existing forests should be prioritized in order to achieve faster recovery.

## Graphical abstract

We examined regeneration patterns of functional composition, functional diversity, and aboveground biomass in a tropical moist secondary forest after 16–22 years since abandonment. We found dissimilar trajectories and recovery status of functional composition, functional diversity indices, and aboveground biomass.

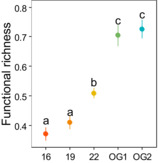

## 1 INTRODUCTION

Agricultural expansion is a major driver of tropical forest loss and the associated loss of biodiversity and ecosystem functions (FAO & UNEP, 2020). About 30%–40% of tropical deforestation during 2000–2010 is attributed to local subsistence agriculture and large‐scale commercial agriculture (FAO & UNEP, 2020). When human‐induced pressures on tropical forests are abandoned, natural regeneration could potentially lead to the development of a secondary forest—that gradually recovers many characteristics of the historical forest (Cramer et al., 2008). Natural regeneration arises from the soil seed bank, rootstocks or stolons present below the soil surface in areas with low levels of soil disturbance, and from seeds dispersed from nearby forests (Martınez‐Ramos et al., 2016). To assist natural regeneration, the abandoned areas are protected from recurring fires, livestock grazing, and invasive species that can hinder tree establishment (Shono et al., 2007). Given the increasing interest in assisted natural regeneration as a restoration strategy (Martınez‐Ramos et al., 2016), secondary forests have become important avenues for recuperating some of the historical biodiversity and ecosystem services (Letcher & Chazdon, 2009).

The increasing proportion of secondary forests necessitates understanding their potential to recover predisturbance biodiversity and ecosystem functioning. To accurately assess the extent of forest recovery (i.e., restoration success), multiyear monitoring is critical (D'Astous et al., 2013; Verhagen et al., 2001). In this case, recovery is assessed in a trajectory analysis, where the impact of restoration is assessed through time by evaluating the distance between the biodiversity and ecosystem functioning of the secondary and old‐growth reference forest (Young et al., 2005). However, most studies on forest recovery (e.g., Letcher & Chazdon, 2009; Lohbeck et al., 2013; Manuel et al., 2018) rely on a static chronosequence approach (i.e., space‐for‐time substitution), which may be confounded by local site variations in environmental conditions and disturbance history (Johnson & Miyanishi, 2008).

Traditionally, trajectory analysis has relied on species diversity and composition, and forest structure as indicators of recovery (Letcher & Chazdon, 2009; Manuel et al., 2018; Oberleitner et al., 2021). However, a narrow focus on taxonomic and structural changes may mask functional shifts that underlie community assembly and ecosystem functioning during forest recovery (Berenguer et al., 2018). The use of functional trait composition and functional diversity is being increasingly advocated for evaluating restoration interventions, as traits are useful in characterizing community development over time (D'Astous et al., 2013; Lohbeck et al., 2012). Since traits determine the ability of a species to establish and persist under a given set of environmental conditions (Lavorel et al., 2010), a trait‐based approach can help clarify community assembly dynamics (D'Astous et al., 2013; Rosenfield & Müller, 2020). For instance, dispersal mode and seed/fruit size dictate a species' ability to colonize and persist in a particular site by influencing the arrival of new plants (Forget et al., 2005). The light requirement of a species (e.g., shade tolerance) affects its survival under closed canopies (Valladares & Niinemets, 2008). Functional diversity expresses the extent of differences among species traits in multidimensional space (Mouchet et al., 2010; Villéger et al., 2008). High functional diversity can result in greater ecosystem resilience to disturbance and higher levels of ecosystem functioning (Cadotte et al., 2011; Montoya et al., 2012).

Assessing the regeneration trajectories of functional composition, functional diversity, and structural parameters like biomass provides a broader understanding of the extent of forest recovery and improves decision‐making for restoration (Berenguer et al., 2018). Previous studies showed that functional composition shifts from dependence on high‐light environments to shade tolerance with time during forest regeneration as light becomes a limiting factor due to canopy closure (Bauters et al., 2018; Montgomery & Chazdon, 2001). In tropical moist forests, early‐colonizing species tend to have lower wood density than late‐colonizing species (Fernandes Neto et al., 2019), and maximum height increases with forest age (Bauters et al., 2018). Cruz‐Alonso et al. (2019) showed that functional diversity increased with age, while Lohbeck et al. (2012) showed no significant influence of age on functional diversity. Aboveground biomass increased with age in secondary tropical forests of Costa Rica (Letcher & Chazdon, 2009; Oberleitner et al., 2021), Brazilian Amazon (Lennox et al., 2018), and Uganda (Omeja et al., 2012). Also, literature shows that functional composition, functional diversity, and aboveground biomass may exhibit altered developmental trajectories due to the presence of undesirable species (like alien invasive species) that compete with native species (Catterall, 2016; Cordell et al., 2016; Tymen et al., 2016) or attract large herbivores like elephants that browse and trample young trees as in Kibale National Park in Uganda (Lawes & Chapman, 2006).

The extent of forest recovery is influenced by landscape factors like the distance to forests, that is, old‐growth forests or forest fragments (Boukili & Chazdon, 2016; Duncan & Duncan, 2000). Increasing distance to forests increases dispersal and microsite limitations to forest regeneration (Duncan & Duncan, 2000; Wijdeven & Kuzee, 2000). Dispersal limitations hinder seed dispersal by animals as most animals, particularly large frugivores dispersing large‐fruited species, will not visit deforested areas due to exposure to predators and hunting (Holl et al., 2000; Wright, 2010). Microsite limitations inhibit tree establishment and growth in restoration sites because at longer distances from forests, the microsite conditions (e.g., lower humidity and higher temperatures) are less favorable for plant growth (Duncan & Duncan, 2000; Tabarelli et al., 2008). The influence of distance to forests on regeneration has received ample theoretical attention from the perspective of taxonomic recovery (Hooper et al., 2005; Oberleitner et al., 2021), but there are limited efforts for functional composition, functional diversity, and ecosystem functioning (Boukili & Chazdon, 2016).

Here, we investigated the regeneration trajectories of tree functional composition, functional diversity, and aboveground biomass in a tropical forest landscape. Specifically, we examined the temporal changes and recovery of functional composition, functional diversity, and aboveground biomass in a secondary forest. We evaluated the role of potential dispersal and microsite limitations by assessing the influence of distance to forests on regeneration. We predicted that (i) functional composition, functional diversity, and aboveground biomass would shift directionally with forest age due to changing environmental conditions during forest regeneration (Boukili & Chazdon, 2016) in accordance with environmental filtering (Lebrija‐Trejos et al., 2010); and (ii) the proportion of forest‐dependent and biotically dispersed species and aboveground biomass would decline with increasing distance to forests due to seed and microsite limitations to tree growth (Duncan & Duncan, 2000; Tabarelli et al., 2008; Wijdeven & Kuzee, 2000).

## 2 MATERIALS AND METHODS

### 2.1 Study area

Our study focused on the secondary and old‐growth moist tropical forests located in the southern part of Kibale National Park (795 km^2^) in western Uganda (00°13′–00°41′N, 30°19′–30°32′E; Omeja et al., 2011). The park receives an average annual rainfall of 1750 mm, and the mean monthly temperature range is 20.8–22.1°C. The elevation is about 900 m in the south to 1590 m a.s.l. in the north. The park has one of the highest concentrations of primates in Africa and supports many other species of mammals, besides reptiles and birds. The old‐growth forest is moist semideciduous with *Fabaceae*, *Ulmaceae*, *Sapotaceae*, *Annonaceae*, and *Apocynaceae* as the dominant tree families (Omeja et al., 2011). The secondary forest is as a result of assisted natural regeneration in former agricultural areas. In 1971, agricultural encroachers cleared about 120 km^2^ of forests in the southern part of the park (Chapman & Lambert, 2000). In 1992, agriculture was abandoned and the formerly encroached areas were dominated by elephant grass (*Cenchrus purpureus*) because recurrent fires set by poachers and livestock keepers prevented sufficient natural regeneration (Omeja et al., 2011). In 1995, Uganda Wildlife Authority (UWA) and Face the Future, a Dutch nongovernmental organization, started restoring forests as carbon offsets using assisted natural regeneration (Emmer, 1998). Assisted natural regeneration encompassed protecting the degraded areas (about 2593 ha) against tree cutting and livestock grazing by conducting regular patrols, fires by controlling poaching, constructing fire lines and monitoring fire incidences by using watch towers, and killing alien species like *Eucalyptus* species through debarking. The secondary forest lies between areas restored using conventional reforestation methods involving planting of tree seedlings and wildlings, to the west and the old‐growth forest to the east.

### 2.2 Experimental design

**FIGURE 1:**
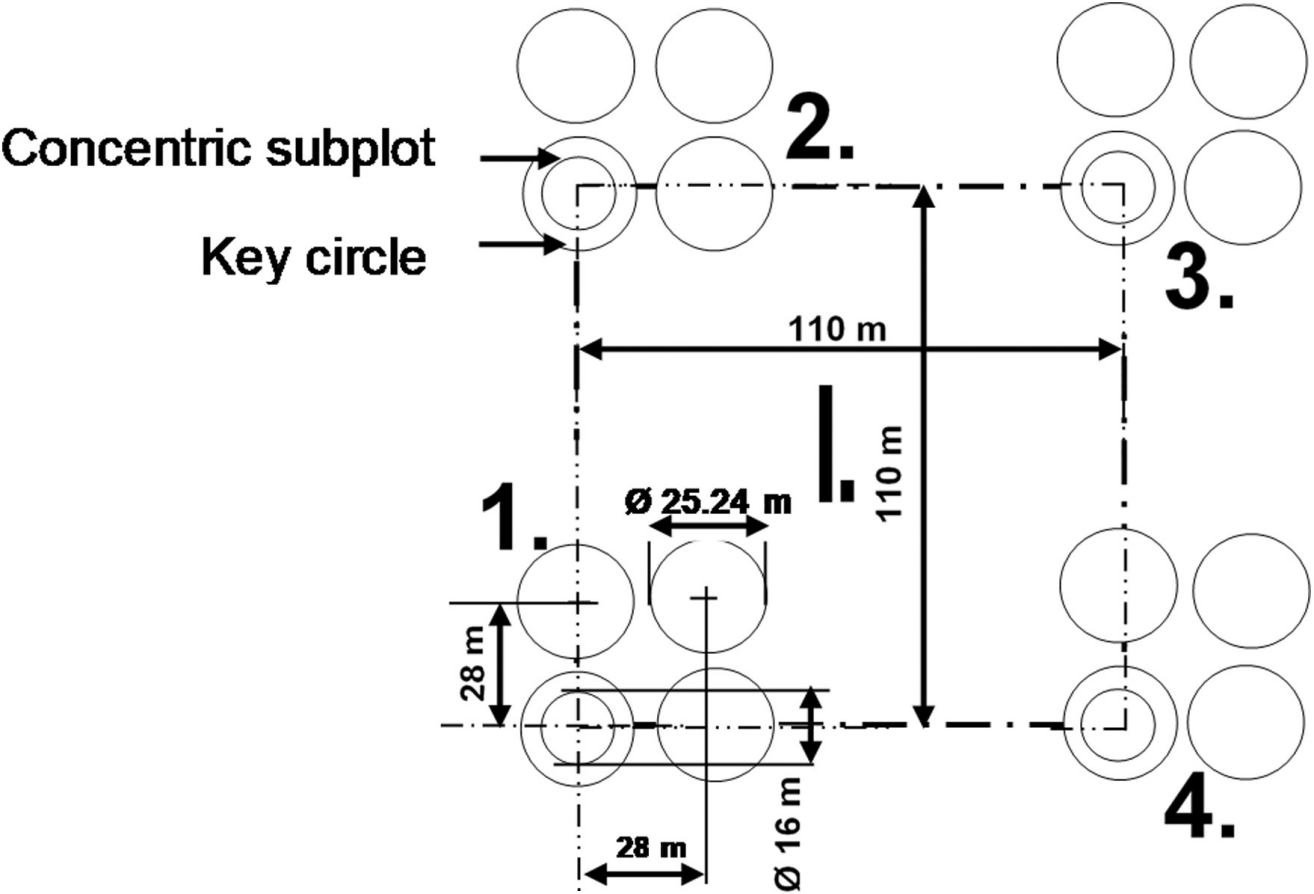
Monitoring cluster (I) consisted of four permanent sample plots (2000 m^2^ each) in the old‐growth forest and three permanent sample plots in the secondary forest in Kibale National Park, Uganda. Each plot was composed of four 500 m^2^ circles. Each key circle contained a concentric subplot.

A regular sampling grid consisting of clusters of four permanent sample plots with a spacing of 500 × 500 m (Figure 1) was applied to two sites in the old‐growth forest (IFER, 2014). The same grid consisting of clusters of three permanent sample plots was applied to four sites in the secondary forest using Field‐Map technology (IFER, 2014). Each sample plot (2000 m^2^) consisted of four 500 m^2^ circles, that is, one key circle at the bottom left of each plot, and three other circles (Figure 1). The key circle contained a 201.1 m^2^ concentric subplot (Figure 1). Overall, two sites, three clusters, and five plots were sampled in the old‐growth forest and four sites, 21 clusters, and 63 plots in the secondary forest.

### 2.3 Tree inventory

The secondary forest was 16, 19, and 22 years old during the first, second, and third inventory, respectively. Tree inventory was carried out in 2011, 2014, and 2017 during the same period (February–May) for each year. Trees of diameter at breast height (DBH, at 1.3 m) ≥5 cm were recorded in the concentric subplot; DBH ≥15 cm in the entire key circle and DBH ≥30 cm in the remaining three circles (Figure 1). The plot sizes and tree size classes in this study are specified for carbon monitoring projects in Uganda (IFER, 2011). All qualifying trees were mapped using Field‐Map and identified to species level following Katende et al. (1995) and Eggeling (1940). Tree diameter was measured using an electronic caliper at breast height (1.3 m), unless there were irregularities at this height or trees were shorter. For individuals with buttresses or other stem irregularities at breast height, DBH was measured above the buttresses or stem irregularities. Tree height was remotely measured using Field‐Map. The distance to forests (the closest boundary of the old‐growth forest or forest fragment) to sample plots in the secondary forest was estimated using local area maps and confirmed by UWA staff.

### 2.4 Functional composition and functional diversity

We studied four categorical traits (i.e., habitat type, dispersal mode, fruit size, and successional group) and two continuous traits (i.e., wood density and maximum height; Table 1). The traits illustrate different ecological processes. Habitat type shows optimum species distribution in relation to environmental gradients (i.e., ecological performance, D'Astous et al., 2013). Dispersal mode influences a species ability to reach suitable sites for germination (Forget et al., 2005). Fruit size is associated with either plant survival or reproductive success (D'Astous et al., 2013; Moles et al., 2003). Successional group determines a species ability to establish and grow in a particular site. Wood density and maximum height are related to growth strategy, stress tolerance, and maximum carbon storage (Chave et al., 2009; Gibert et al., 2016; Rosenfield & Müller, 2020).

**TABLE 1:** Description of functional traits (habitat, dispersal, fruit size, successional groups, wood density, and maximum height) of tree species in the secondary and old‐growth forest in Kibale National Park, western Uganda.

Traits	States	Description	Reference
Habitat	Open habitat	Occurs in woodland, grassland, rocky places, bush/thickets or swamp	Lwanga (1996)
	Forest‐dependent	occurs in forest interior, edge, and/or riverine forest	
	Forest nondependent	Occurs in at least one of the open habitats and at least one of the forested habitats	
Dispersal	Biotic	Dispersed by animals such as birds and mammals	Eggeling (1940)
	Abiotic	Dispersed by wind or other abiotic vector	
Fruit size	Small‐fruited	<1 cm	Babweteera and Ssekuubwa (2017)
	Medium‐fruited	1–3 cm	
	Large‐fruited	3.1 to >5.0 cm	
Successional group	Pioneer	Unable to establish in closed forest shade	Fauset et al. (2012)
	Nonpioneer light demander	Seedlings are present in the understory but require higher light environments to reach adult size	
	Shade‐tolerant	Able to establish in closed forest shade	
Wood density	Values in g/cm^3^	Species specific or family level	Fauset et al. (2012)
Maximum height	Values in m	Maximum adult height	Vargas‐larreta et al. (2021)

Information on the categorical traits of species was obtained from literature sources from Uganda (i.e., Eggeling, 1940; Katende et al., 1995; Lwanga, 1996). Species wood density was obtained from the Tree Functional Attributes and Ecological Database (TFAED; Harja et al., 2019) and the African Wood Density Database (AWDD; Carsan et al., 2012) using genus or family averages in case species‐level information was not available (Fauset et al., 2012). Species‐ and family‐level values were used for 95% and 5% of stems, respectively. Fruit size was based on maximum length or diameter, whichever was bigger (Babweteera & Ssekuubwa, 2017). Maximum tree height for each species was extracted from the inventory data. For all the 124 species sampled, we were able to gather all traits for 123 species, that is, 98% of the community (Table S1).

We used community‐weighted mean (CWM) of each trait as a measure of functional composition. CWM describes the dominant functional trait value of the overall community by weighting species trait values by species abundance (Lavorel et al., 2008). The CWM was calculated using the trait value per species in each plot (Garnier et al., 2004). We calculated distances between species in trait space using Gower dissimilarity (*gowdis* function in *FD* package; Laliberté et al., 2014; R Core Team, 2020) and determined CWM and functional diversity for each plot and each inventory year using the *dbFD* function in FD package (Laliberté et al., 2014; R Core Team, 2020). Only 13 individuals belonged to open habitat (woodland), and its CWM was zero in the secondary and old‐growth forest so it was not included in further analyses.

We calculated five complementary measures of functional diversity, that is, functional richness, functional evenness, functional dispersion, functional divergence, and Rao's quadratic entropy (RaoQ's entropy). Functional richness represents the range of traits in a community quantified by the volume of trait space (Villéger et al., 2008). Functional evenness summarizes how species' abundances are distributed throughout the trait space occupied. Functional divergence describes the variation in trait values, weighted by the abundance of each species in a community (Mason et al., 2013). Functional dispersion indicates the distribution of abundances in trait space relative to an abundance‐weighted centroid and the volume of space occupied (Laliberte & Legendre, 2010). RaoQ's entropy estimates the abundance‐weighted variance of the dissimilarities between all species pairs (Botta‐Dukát, 2005).

### 2.5 Biomass estimates

We calculated aboveground biomass of individual stems using a general allometric equation, based on tree DBH (cm), height (*H*, m), and wood density (*ρ*, g/cm^3^) (Chave et al., 2005) obtained from TFAED (Harja et al., 2019) and AWDD (Carsan et al., 2012) of each species, using genus or family averages whenever species‐level information was not available (Fauset et al., 2012). Aboveground biomass was calculated as follows:

$$\text{Aboveground Biomass}=0.0673\;{\left(\rho {\mathrm{DBH}}^{2}H\right)}^{0.976}$$

The aboveground biomass of each stem was calculated in megagrams (Mg) and divided by the subplot area in hectares (ha) to obtain aboveground biomass (Mg/ha) of every stem. The total aboveground biomass per plot was the sum of the aboveground biomass of all stems in the subplots.

### 2.6 Statistical analysis

Statistical analysis was done in R (R Core Team, 2020). To assess the completeness of tree inventory in the secondary and old‐growth forest, we generated sample‐based species accumulation curves using iNEXT package (Chao et al., 2014).

#### 2.6.1 Variation of functional composition, functional diversity, and aboveground biomass with forest age

To determine how CWM values (i.e., functional composition), functional diversity, and aboveground biomass vary with forest age and evaluate restoration success, we fitted separate linear mixed‐effects models with a Gaussian distribution for CWM of each trait, functional diversity indices, and aboveground biomass as response variables (Pinheiro et al., 2014). Each model included forest age as a five‐level factor variable, that is, 16, 19, and 22 years (age when the secondary forest was inventoried in 2011, 2014, and 2017) as well as OG1 and OG2 (for the old‐growth values measured in 2011 and 2017 as reference values) as the explanatory variable. We kept data from different inventory years in the secondary and old‐growth forest separate to control for temporal pseudoreplication. Each model contained random effects; plot nested within cluster nested within site to control for residual variation due to idiosyncratic local site factors. We conducted multiple comparisons between age categories with Tukey's HSD test. Following Zuur et al. (2013), we carried out a graphical evaluation of the standardized residuals to ensure compliance with homogeneity (residuals vs. fitted values), normality (quantile‐quantile plots), and independence (residuals vs. each explanatory variable) assumptions.

#### 2.6.2 Variation of functional composition, functional diversity, and aboveground biomass with distance to forests

Similar models as in 2.6.1 were fitted with distance to forests, a continuous variable, as a fixed effect (Pinheiro et al., 2014). We also included the interaction between forest age and distance to forests as a fixed factor to determine whether the effect of distance varied with forest age. We selected the most parsimonious model by backward selection using Akaike Information Criteria (Burnham & Anderson, 2002). The statistical significance of distance was tested using the Likelihood Ratio Test (LRT, *χ* ^2^) (Zuur et al., 2013).

## 3 RESULTS

**FIGURE A1:**
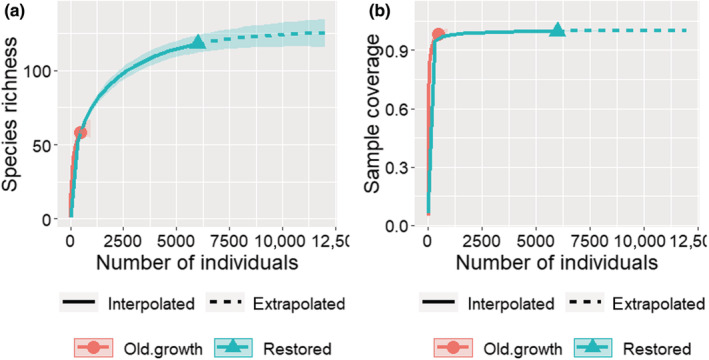
Species accumulation curves for treeinventory in the regrowth and old‐growth forests in Kibale National Park,Uganda. Sample‐size‐based rarefaction(solid line segment) and extrapolation (dotted line segments) curves (a) with95% confidence intervals (shaded areas) and Sample completeness curves (b) forthe tree inventory data.

We recorded 2545 trees belonging to 124 plant species in the secondary and old‐growth forest (Table S1). *Mimusops kummel* with 30 individuals was the most abundant species in the old‐growth forest, and *Shirakiopsis elliptica* with 344 individuals was the most abundant in the secondary forest (Table S1). We found 69 species restricted to the secondary forest and 27 species restricted to the old‐growth forest. Forty species were represented by only one individual. The species accumulation curves showed that the sampling effort was adequate for the secondary forest but not the old‐growth forest (Figure A1).

### 3.1 Variation of functional composition, functional diversity, and aboveground biomass with forest age

**FIGURE 2:**
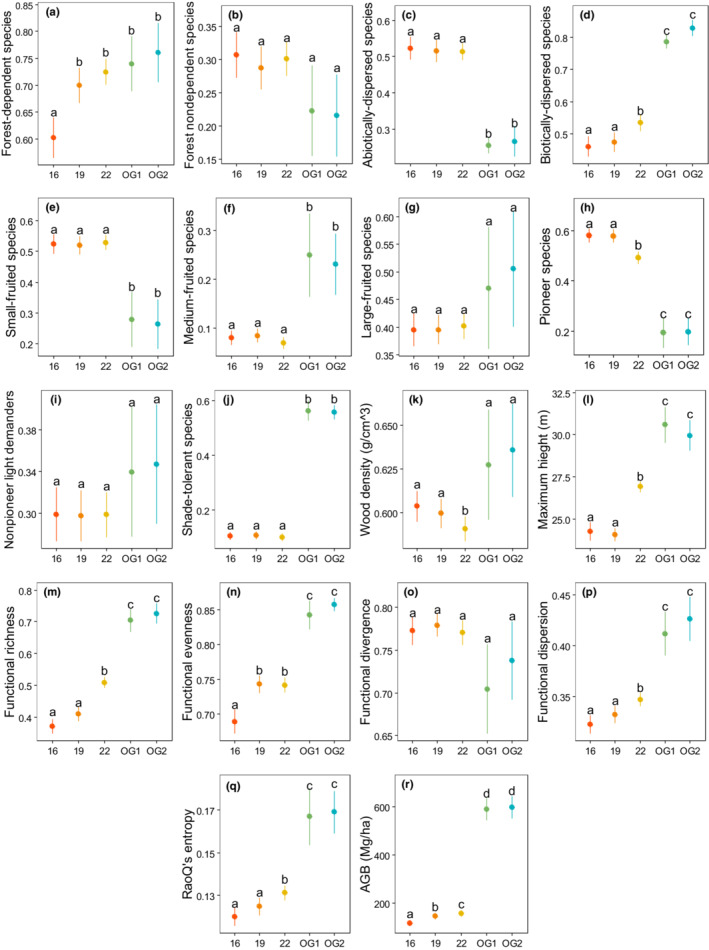
Variation of community‐weighted mean of functional traits (a–l), functional diversity indices (m–q), and aboveground biomass (r) with forest age in Kibale National Park, Uganda. Forest age includes 16, 19, and 22 years when the secondary forest was sampled in 2011, 2014, and 2017, respectively, and OG1 and OG2, which are reference age categories for old‐growth forest samples in 2011 and 2017.

The results showed dissimilar trajectories of functional composition, functional diversity, and aboveground biomass of the secondary forest (Figure 2, Tables A1 and A2). The CWM of forest‐dependent species changed directionally toward the old‐growth forest, being significantly higher at 19 and 22 years than at 16 years (Figure 2a). There was no significant variation in CWM of forest nondependent species with forest age (Figure 2b). Unlike the CWM of abiotically dispersed species (Figure 2c), the CWM of biotically dispersed species increased with forest age (Figure 2d). We found that CWM of fruit sizes in the secondary forest did not respond to forest age; small‐fruited species (Figure 2e), medium‐fruited species (Figure 2f), and large‐fruited species (Figure 2g). The CWM of pioneer species reduced with forest age and was significantly lower at 22 years than at 16 and 19 years (Figure 2h). We found no significant variation with forest age for CWM of nonpioneer light demanders (Figure 2i) and shade‐tolerant species (Figure 2j). The CWM of wood density reduced (Figure 2k) and CWM of maximum height increased (Figure 2l) with forest age.

Among the functional diversity indices, functional richness (Figure 2m), evenness (Figure 2n), dispersion (Figure 2p), and RaoQ's entropy (Figure 2q) increased with forest age toward the old‐growth forest. The trajectory of functional divergence (Figure 2o) did not change significantly with forest age. Aboveground biomass increased significantly over time toward the old‐growth forest (Figure 2r). Generally, the old‐growth forest showed no significant changes in functional composition, functional diversity, and aboveground biomass during the sampling period (Figure 2).

### 3.2 Restoration success

We considered restoration success as the absence of significant differences in functional composition, functional diversity, and aboveground biomass between the secondary and old‐growth forest. At 19 and 22 years, the secondary forest reached similar CWM of forest‐dependent species (Figure 2a) and had similar CWM of forest nondependent species at 16, 19, and 22 years (Figure 2b) as the old‐growth forest (Table A2). The secondary forest consistently showed significantly higher CWM of abiotically dispersed species (Figure 2c) and lower CWM of biotically dispersed species at 16, 19, and 22 years than the old‐growth forest (Figure 2d). The secondary forest had significantly higher CWM of small‐fruited (Figure 2e), lower CWM of medium‐fruited (Figure 2f) and similar CWM of large‐fruited species (Figure 2g) compared with the old‐growth forest. The secondary forest exhibited significantly higher CWM of pioneer species (Figure 2h), similar CWM of nonpioneer light demanders (Figure 2i) and lower CWM of shade‐tolerant species at 16, 19, and 22 years (Figure 2j) in comparison with the old‐growth forest. The secondary forest at 16 and 19 years had similar CWM of wood density as the old‐growth forest, but at 22 years, the secondary forest showed lower CWM of wood density (Figure 2k). Also, the secondary forest had significantly lower CWM of maximum height than the old‐growth forest (Figure 2l).

Among the functional diversity indices, functional richness (Figure 2m), functional evenness (Figure 2n), functional dispersion (Figure 2p), and RaoQ's entropy (Figure 2q) of the secondary forest were consistently lower at 16, 19, and 22 years than the old‐growth forest. However, functional divergence was not significantly different between the secondary and old‐growth forest (Figure 2o). The secondary forest showed significantly lower aboveground biomass at 16, 19, and 22 years than the old‐growth forest, recuperating on average 24% of 600 Mg/ha of the biomass in the old‐growth forest (Figure 2r).

### 3.3 Variation of functional composition, functional diversity, and aboveground biomass with distance to forests

**FIGURE 3:**
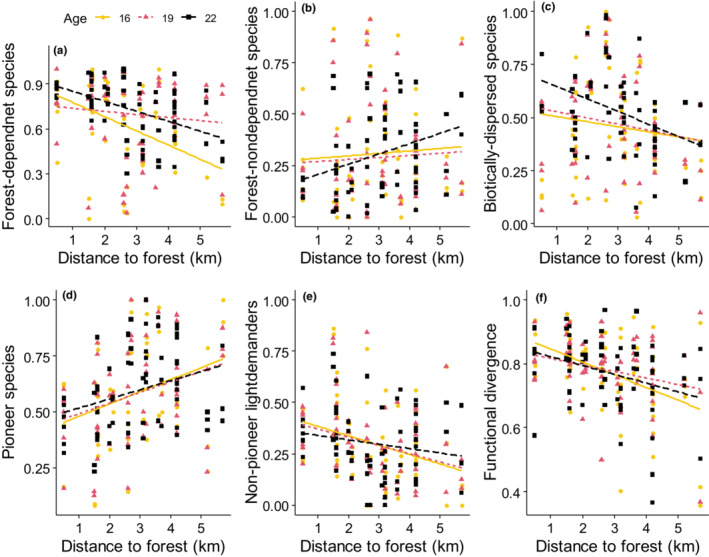
Variation of functional composition (a–e) and functional diversity, that is, divergence (f) with distance to forests in Kibale National Park, Uganda in 2011, 2014, and 2017.

The CWM of forest‐dependent species in the secondary forest declined (Figure 3a) while CWM of forest nondependent species increased (Figure 3b) with increasing distance to forests (Table 2, Table A3). The CWM of biotically dispersed species (Figure 3c) declined significantly while CWM of abiotically dispersed species did not vary significantly with distance to forests (Table 2). The effect of distance to forests on forest‐dependent and biotically dispersed species tended to decline with time (Figure 3). The CWM of small‐fruited species, medium‐fruited species, and large‐fruited species was not significantly affected by distance to forests (Table 2). Increasing distance to forests increased the CWM of pioneer species (Figure 3d) and reduced CWM of nonpioneer light demanders (Figure 3e) but did not significantly affect CWM of shade‐tolerant species (Table 2). The distance to forests did not significantly affect CWM of wood density and maximum height (Table 2). Increasing distance to forests reduced functional divergence (Figure 3F, Table 2). There was no significant variation of functional richness, evenness, dispersion, and RaoQ's entropy with distance to forests (Table 2). Aboveground biomass did not vary significantly with distance to forests (Table 2).

**TABLE 2:** Likelihood ratio test (*χ* ^2^, *df* = degrees of freedom) for the variation of functional composition (community‐weighted mean—CWM), functional diversity, and aboveground biomass in the secondary forest with distance to forests in Kibale National Park, western Uganda.

Attribute	*χ* ^2^	*df*	*p*
CWM of forest‐dependent species	11.30	2	.004
CWM of forest nondependent species	6.84	2	.032
CWM of abiotically dispersed species	0.35	1	.557
CWM of biotically dispersed species	5.43	2	.046
CWM of small‐fruited species	0.74	1	.339
CWM of medium‐fruited species	0.30	1	.583
CWM of large‐fruited species	0.75	1	.386
CWM of pioneer species	9.65	1	.002
CWM of nonpioneer light demanders	3.88	1	.049
CWM of shade‐tolerant species	1.43	1	.231
CWM of wood density (g/cm^3^)	1.59	1	.207
CWM of maximum height (m)	0.94	1	.332
Functional richness	0.15	1	.696
Functional evenness	1.56	1	.212
Functional divergence	10.19	1	.001
Functional dispersion	0.51	1	.474
RaoQ's entropy	0.52	1	.473
Aboveground biomass (Mg/ha)	0.84	1	.360

*Note*: Values are significant at *p* < .05. Table A3 shows the results of the most parsimonious mixed‐effects model.

## 4 DISCUSSION

In this study, we examined the trajectory of a secondary forest restored through assisted natural regeneration and assessed restoration success from the perspective of functional composition, functional diversity, and aboveground biomass of tree communities. We also determined how these attributes are influenced by distance to forests (old‐growth forests or forest fragments). The secondary forest showed dissimilar trajectories and recovery status of the functional attributes and aboveground biomass. Increasing distance to forests reduced the CWM of forest‐dependent species, biotically dispersed species and nonpioneer light demanders, and functional divergence but increased the CWM of forest nondependent and pioneer species. However, the findings on restoration success should be interpreted with caution because the sample‐based species accumulation curves showed that the old‐growth forest may harbor more species than actually sampled. Besides, our statistical analyses might be influenced by the unbalanced study design arising from unequal sampling in terms of plots (i.e., 63 in the secondary vs. five in the old‐growth forest) and sampling years (i.e., 2011, 2014, 2017 for the secondary vs. 2011 and 2017 for the old‐growth forest).

### 4.1 Variation of functional composition, functional diversity, and aboveground biomass with forest age

Consistent with our first prediction that functional composition of the secondary forest would shift directionally with forest age, we found a significant increase in CWM of forest‐dependent species and biotically dispersed species with forest age. We attribute the increase in forest‐dependent species to the improvement of microsites due to environmental filtering as the forest develops (Boukili & Chazdon, 2016). The forest‐dependent species in this study included pioneers, nonpioneer light demanders, and shade‐tolerant species. Initially, at the beginning of restoration, it is likely that forest‐dependent pioneer species arising from the soil seed bank or dispersed by wind or disturbance‐adapted bats and small birds colonize the degraded sites (Estrada‐Villegas et al., 2022; Palma et al., 2021). These in addition to remnant trees, modulate environmental filters such as light, water, and nutrient availability, and facilitate regeneration of forest‐dependent late successional species (nonpioneer light demanders and shade‐tolerants) as the forest develops (Jacob et al., 2017). Therefore, the early‐ and late‐colonizing species could lead to an increase in the CWM of forest‐dependent species as regeneration progresses. The observed increase with age in the CWM of forest‐dependent species suggests that the secondary forest is on a trajectory toward the old‐growth forest.

The increase in CWM of biotically dispersed species reported here could suggest increased attractiveness of the secondary forest to animals such as birds. The early successional species and remnant trees provide perching sites, feeding sites for fruits and invertebrate prey and nest sites for both passing and resident birds (Griscom & Ashton, 2011; Jacob et al., 2017). The frugivorous birds drop or regurgitate seeds and fruits which fall under the canopies of remnant trees during their stay, thus facilitating seed dispersal (Griscom & Ashton, 2011; Jacob et al., 2017). If we assume that the dispersed seeds survive and germinate due to modulation of environmental filters as the forest develops, they could establish in the secondary forest thereby increasing the proportion of biotically dispersed species as the forest ages. Previous studies also reported an increase with forest age in the dependence on biotic dispersal (Lohbeck et al., 2013).

The CWM of pioneer species declined with forest age, which also corroborates our first prediction. Pioneer species thrive under high‐light environments in young secondary forests (Lohbeck et al., 2013). As the forest develops, light becomes a limiting resource in the understory of moist forests (Denslow & Guzman, 2000; Montgomery & Chazdon, 2001), which potentially leads to a decline in the CWM of pioneer species. The CWM of wood density declined with forest age which was not expected as previous studies showed that wood density increases due to an increase in shade tolerance as the forest develops (Valladares & Niinemets, 2008). Since certain highly abundant pioneers in our study have high wood density (e.g., *Diospyros mespiliformis*; wood density = 0.98, *Diospyros abyssinica*; wood density = 0.79 and *Celtis africana*; wood density = 0.73), the decline in CWM of wood density may be linked to a decline in CWM of pioneers with forest age as reported by this study. In addition, some high wood density species introduced by illegal occupants (e.g., *Eucalyptus saligna*, wood density = 0.83), and native trees left standing when the forest was cleared (Jacob et al., 2017) or species able to survive repeated disturbances like fire (e.g., *Thevetia peruviana*; wood density = 0.72 and *Phoenix reclinata*, wood density = 0.74), are present early in forest regeneration. In this regard, the absence of the expected CWM wood density pattern might indicate a historical contingency related to land use (Muscarella et al., 2017).

The increase in CWM of maximum height with forest age may also be linked to the improvement in microsites during forest regeneration. The observed increase in maximum height implies that the secondary forest is shifting toward a higher dominance of tall species as in the old‐growth forest (Ruiz‐Benito et al., 2017) and suggests an increase in structural complexity (i.e., presence of emergent species and different strata) as a result of restoration, which could benefit forest‐dependent animals by enhancing habitat availability (Staples et al., 2020).

Also, we revealed an increase in functional richness, evenness, dispersion, and RaoQ's entropy with forest age. The increase in functional richness could indicate an increase in the recruitment of species that occupy vacant functional niches created as a result of habitat enhancement by restoration (Murphy et al., 2016). The progressive increase in functional evenness reflects an increase in the relative contribution by different species to an observed community‐level process (Chao & Ricotta, 2019) and an increase in functional redundancy, thereby making the ecosystem function of the secondary forest resilient to species extinction (Naeem, 1998; Rosenfeld & Mall, 2002). Increasing functional dispersion increases response diversity (i.e., variability of responses to habitat enhancement among species that contribute similarly to ecosystem functioning; Elmqvist et al., 2009), which may enhance the resilience of communities to human and natural disturbances. The increase in RaoQ's entropy may increase the dissimilarities among species (Botta‐Dukát, 2005), which reduces competition and increases complementarity resource use (Diaz & Cabido, 2001). By contrast, there was no significant effect of forest age on functional divergence, which demonstrates that different functional diversity measures may vary in their response to forest restoration, and highlights the need to include multiple functional diversity measures in studies evaluating the efficacy of restoration in highly diverse ecosystems like tropical forests.

Aboveground biomass of the secondary forest increased significantly with forest age, which supports the findings of previous studies (Aide et al., 2000; Oberleitner et al., 2021; Staples et al., 2020). This observation may be linked to the positive effect of forest age on tree diameter (*χ* ^2^ = 43.61, *df* = 1, *p* < .001) and height (*χ* ^2^ = 51.49, *df* = 1, *p* < .001)—the structural attributes used to compute aboveground biomass in this study. The increase in these structural attributes could be linked to the temporal development of vegetation during forest regeneration. Since aboveground biomass provides an index of carbon sequestration (as forests store)—principally in the form of plant biomass (Aguiar et al., 2016)—37% of the planet's terrestrial carbon (U.S. DOE, 2010) and is the major driver of changes in ecosystem processes (Lohbeck et al., 2018), the positive effect of forest age on aboveground biomass implies that regeneration increases forest carbon stocks (Letcher & Chazdon, 2009; Oberleitner et al., 2021) and re‐establishes biological control over ecosystem processes (Lohbeck et al., 2018).

### 4.2 Restoration success

We found no significant difference in CWM of forest‐dependent species, forest nondependent species, large‐fruited species, and nonpioneer light demanders, implying recovery of these functional attributes within 16–22 years since abandonment. However, the secondary forest had higher CWM of abiotically dispersed, small‐fruited, and pioneer species than the old‐growth forest. This observation could be attributed to the secondary forest being relatively more open than the old‐growth forest, thereby supporting pioneer species with many small seeds that can travel long distances by wind (Lohbeck et al., 2013; Muscarella et al., 2017). The CWM of biotically dispersed species was significantly lower in the secondary than old‐growth forest, which suggests that a full recovery of biotic dispersal might take longer than 22 years, for instance 40–50 years in central Panama (Estrada‐Villegas et al., 2022). Since the ability of animals to colonize restored areas depends on vegetation structure (Elliot Noe et al., 2022), we predict that full recovery of the secondary forest structure (i.e., tree height and aboveground biomass) might facilitate recovery of biotic dispersal.

There was lower CWM of medium‐fruited species in the secondary than old‐growth forest. It is unlikely that there are dispersal limitations to medium‐fruited species since species which disperse large‐fruited species that were similar between the forest types can also disperse medium‐fruited species (Albrecht et al., 2018). Given that seed arrival does not guarantee seedling recruitment and tree establishment (Reid & Holl, 2013) and seed predation within our study area is greater in naturally regenerating secondary forests than in old‐growth forests (Ssekuubwa et al., 2018), we attribute the lower CWM of medium‐fruited species in the secondary forest to selective seed predation on medium‐fruited species like *Diospyros mespiliformis*, *Dombeya mukole*, *Aphania senegalensis*, and *Phoenix reclinata*.

The CWM of shade‐tolerant species was significantly lower in the secondary than old‐growth forest which we attribute to the secondary forest being a relatively open habitat with a simpler vegetation structure, yet shade‐tolerant species thrive under shade (Poorter et al., 2019). The CWM of wood density of the secondary forest at 22 years was significantly lower than in the old‐growth forest, which may be linked to the lower CWM of shade‐tolerant species (Muscarella et al., 2017; Poorter et al., 2019). Besides, it is likely that at 22 years, there is only a limited subset of pioneer species with high wood density since this study has also shown a general decline in CWM of pioneer species. The lower CWM of shade‐tolerant species and wood density suggest that the secondary forest is dominated by species with acquisitive traits, which contrasts the functional composition in the old‐growth forest with conservative traits (Muscarella et al., 2017; Poorter et al., 2019).

The CWM of maximum height was significantly lower in the secondary forest than in the old‐growth forest. Our result suggests that more time is needed for the secondary forest to attain higher dominance of tall species (Ruiz‐Benito et al., 2017), as it is not yet a suitable habitat for some old‐growth specialists with the greatest heights (e.g., *Trilepisium madagascariense*, height = 50 m, *Monodora angolensis*, height = 46 m, *Aningeria altissima*, height = 42 m), which should be expected as we observed a significant increase in maximum height as the forest ages. Since height is a measure of a species competition ability (Ruiz‐Benito et al., 2017), the shorter height in the secondary forest suggests lower competition for light, water, and nutrients.

Despite the fast recovery of functional divergence, there were still important functional diversity differences as functional richness, evenness, dispersion, and RaoQ's entropy were lower in the secondary than old‐growth forest. Our results suggest that full recovery of functional diversity of secondary forests might take much longer than 22 years. Maeshiro et al. (2013) found similar recovery patterns of functional richness, evenness, and divergence for tree communities in 40‐year‐old subtropical secondary forests on Ryukyu Island in Japan. Previous studies attributed higher functional diversity in reference ecosystems to natural heterogeneity usually found in mature ecosystems (Bartels & Chen, 2010; D'Astous et al., 2013).

Within 16–22 years, the secondary forest recovered 24% of the 600 Mg/ha aboveground biomass of the old‐growth forest, which is not consistent with findings from other tropical forests, for instance Letcher and Chazdon (2009) and Staples et al. (2020) found that aboveground biomass recovered fully after 21–30 years in a Costa Rican and 30 years in an Australian tropical secondary forest. The variation in recovery rates may be linked to differences in disturbance history, for example, cultivation in Uganda (Ssekuubwa et al., 2018) and ranching in Costa Rica (Oberleitner et al., 2021), and the generally slower rate of forest succession in Afrotropical than Neotropical forests (Chapman & Chapman, 1996).

### 4.3 Effect of distance to forests

In line with our prediction that the proportion of forest‐dependent species would decline with increasing distance to forests, we observed a decline in CWM of forest‐dependent species which we attribute to unfavorable conditions (e.g., lower humidity and higher temperatures) for plant growth since the ameliorative effect of the old‐growth forest on microsite limitations declines at longer distances (Duncan & Duncan, 2000; Tabarelli et al., 2008). The decline in CWM of biotically dispersed species reported in this study might be a result of reduced zoochorous seed rain since other studies have shown that the proportion of biotically dispersed species in seed rain declines with increasing distance to forests (Martınez‐Ramos et al., 2016). Most animals, particularly large frugivores dispersing large‐fruited tree species may not visit distant open habitats due to a lack of fruit, branches for perching or exposure to predators and hunting (Holl et al., 2000; Wright, 2010). The decline in nonpioneer light demanders with distance to forests could be attributed to the general decline in forest‐dependent species since most nonpioneer light demanders (68%) recorded in this study were also forest‐dependent species. On the contrary, the CWM of forest nondependent and pioneer species increased at longer distances from forests, which was expected as these species thrive in the rich light environment in open areas with lower forest cover (Lohbeck et al., 2013). Among the functional diversity measures, functional divergence declined with increasing distance, which may be attributed to dispersal and microsite limitations on the arrival and establishment of species with different functional traits into restoration sites (Duncan & Duncan, 2000; Mason et al., 2013; Tabarelli et al., 2008; Wijdeven & Kuzee, 2000).

## 5 CONCLUSIONS

We found dissimilar regeneration trajectories of tree functional composition, functional diversity, and aboveground biomass. Overall, the secondary forest is evolving toward the old‐growth forest manifested in an increase with forest age in forest dependence, biotic dispersal, maximum height, functional diversity, and aboveground biomass, and a decline in the proportion of pioneer species. The secondary forest recovered the CWM of forest‐dependent species, forest nondependent species, large‐fruited species, nonpioneer light demanders, and functional divergence after 16–22 years since abandonment. However, the secondary forest had not yet reached equivalent values of most attributes of functional composition, diversity and AGB in the old‐growth forest, which requires continued monitoring of the regeneration trajectories. Increasing distance from forests reduced the proportion of forest‐dependent species, biotically dispersed species and nonpioneer light demanders, functional divergence and increased forest nondependent and pioneer species.

Since the observed patterns of functional attributes and aboveground biomass may be influenced by demographic processes like recruitment, growth, and mortality (Muscarella et al., 2017), future studies should aim to assess the influence of demographic processes on regeneration trajectories. This study focused on aboveground functional traits and biomass, we recommend future studies on belowground attributes, and the influence of soil and climate on regeneration, in order to fully understand the regeneration dynamics in abandoned tropical landscapes. We note that the robustness of our results may be affected by the lower sample effort in the old‐growth forest compared with the secondary forest; nonetheless, we used repeated measures of permanent sample plots, which makes our trajectory analysis reliable (Powers & Kozak, 2019).

## AUTHOR CONTRIBUTIONS

**Enock Ssekuubwa:** Conceptualization (equal); data curation (supporting); formal analysis (lead); investigation (supporting); visualization (lead); writing – original draft (lead); writing – review and editing (equal). **Wouter van Goor:** Data curation (equal); investigation (equal); methodology (equal); project administration (supporting); writing – review and editing (equal). **Martijn Snoep:** Data curation (equal); investigation (equal); methodology (equal); project administration (lead); writing – review and editing (equal). **Kars Riemer:** Data curation (equal); investigation (equal); methodology (equal); project administration (supporting); writing – review and editing (equal). **Fredrick Wanyama:** Data curation (equal); investigation (equal); methodology (equal); project administration (supporting); writing – review and editing (equal). **Daniel Waiswa:** Conceptualization (equal); formal analysis (supporting); writing – review and editing (equal). **Fred Yikii:** Conceptualization (equal); formal analysis (supporting); writing – review and editing (equal). **Mnason Tweheyo:** Conceptualization (equal); formal analysis (supporting); writing – review and editing (equal).

## CONFLICT OF INTEREST STATEMENT

We declare no conflicts of interest.

## Supporting information


Table S1

## Data Availability

The data that support the findings of this study are openly available in the Dryad Digital Repository: https://doi.org/10.5061/dryad.1c59zw3w7 (Ssekuubwa et al., 2021).

## Appendix

**TABLE A1:** Community‐weighted mean (CWM) of functional traits and functional diversity (Mean ± Standard error) of the secondary forest at 16, 19, and 22 years and old‐growth forests in Kibale National Park, Uganda.

Trait	16 years	19 years	22 years	OG1	OG2
Forest‐dependent species	0.60 ± 0.03	0.70 ± 0.03	0.72 ± 0.02	0.74 ± 0.04	0.76 ± 0.03
Forest nondependent species	0.31 ± 0.03	0.29 ± 0.03	0.30 ± 0.03	0.22 ± 0.07	0.22 ± 0.06
Abiotically dispersed species	0.52 ± 0.03	0.52 ± 0.03	0.51 ± 0.02	0.26 ± 0.02	0.27 ± 0.04
Biotic‐dispersed species	0.46 ± 0.03	0.47 ± 0.03	0.53 ± 0.02	0.79 ± 0.02	0.83 ± 0.03
Small‐fruited species	0.52 ± 0.03	0.52 ± 0.03	0.53 ± 0.02	0.28 ± 0.09	0.26 ± 0.08
Medium‐fruited species	0.08 ± 0.02	0.08 ± 0.01	0.07 ± 0.01	0.25 ± 0.09	0.23 ± 0.06
Large‐fruited species	0.40 ± 0.03	0.40 ± 0.03	0.40 ± 0.02	0.47 ± 0.11	0.51 ± 0.10
Pioneer species	0.58 ± 0.03	0.58 ± 0.03	0.49 ± 0.03	0.19 ± 0.06	0.20 ± 0.05
Nonpioneer light demander species	0.30 ± 0.03	0.30 ± 0.02	0.30 ± 0.02	0.34 ± 0.06	0.35 ± 0.06
Shade‐tolerant species	0.11 ± 0.01	0.11 ± 0.01	0.10 ± 0.02	0.56 ± 0.04	0.56 ± 0.03
Wood density (g/cm^3^)	0.60 ± 0.01	0.60 ± 0.01	0.59 ± 0.01	0.63 ± 0.03	0.64 ± 0.03
Maximum height (m)	24.26 ± 0.54	24.06 ± 0.39	26.91 ± 0.32	30.58 ± 1.06	29.96 ± 0.91
Functional richness	0.37 ± 0.02	0.41 ± 0.02	0.51 ± 0.02	0.70 ± 0.04	0.73 ± 0.03
Functional evenness	0.69 ± 0.02	0.74 ± 0.01	0.74 ± 0.01	0.84 ± 0.02	0.86 ± 0.01
Functional divergence	0.77 ± 0.02	0.78 ± 0.01	0.77 ± 0.01	0.70 ± 0.05	0.74 ± 0.05
Functional dispersion	0.32 ± 0.01	0.33 ± 0.01	0.35 ± 0.01	0.41 ± 0.02	0.43 ± 0.02
RaoQ's entropy	0.12 ± 0.004	0.12 ± 0.01	0.13 ± 0.01	0.17 ± 0.01	0.17 ± 0.01
Aboveground biomass (Mg/ha)	116.10 ± 12.20	145.90 ± 13.70	156.40 ± 12.50	587.40 ± 46.20	595.90 ± 45.00

*Note*: The secondary forest was sampled in 16, 19, and 22, while the reference old‐growth forest was sampled in 16 (OG1) and 22 (OG2).

**TABLE A2:** Variation of functional composition (community‐weighted mean—CWM), functional diversity, and aboveground biomass of the secondary and old‐growth forests with time since abandonment in Kibale National Park, Uganda.

Fixed effects	Estimate	SE	*T*	*p*	Estimate	SE	*T*	*p*
	**CWM of forest‐dependent species**				**CWM of shade‐tolerant species**			
Intercept	0.505	0.038	13.38	<.001	0.116	0.035	3.34	.174
Age (19 – 16)	0.187	0.032	5.79	<.001	0.002	0.011	0.20	.841
Age (22 – 16)	0.220	0.032	6.84	<.001	−0.001	0.011	−0.06	.963
Age (OG1 – 16)	0.294	0.125	2.36	.018	0.440	0.077	5.74	<.001
Age (OG2 – 16)	0.327	0.125	2.63	.008	0.188	0.159	1.19	<.001
**Random effects**	**Name**	**Variance**	**SD**		**Name**	**Variance**	**SD**	
Plot: Cluster: Sites	Intercept	0.009	0.094		Intercept	0.005	0.072	
Cluster: Sites	Intercept	0.017	0.129		Intercept	0.002	0.043	
Sites	Intercept	~0	~0		Intercept	0.004	0.061	
	**CWM of forest nondependent species**				**CWM of wood density**			
Intercept	0.559	0.059	9.51	<.001	0.608	0.015	41.22	<.001
Age (19 – 16)	−0.018	0.026	−0.68	.497	−0.004	0.005	−0.79	.427
Age (22 – 16)	−0.009	0.026	−0.35	.724	−0.012	0.005	−2.26	.024
Age (OG1 – 16)	−0.091	0.164	−0.55	.581	0.028	0.037	0.74	.462
Age (OG2 – 16)	−0.096	0.164	−0.59	.558	0.036	0.037	0.96	.338
**Random effects**	**Name**	**Variance**	**SD**		**Name**	**Variance**	**SD**	
Plot: Cluster: Sites	Intercept	0.039	0.197		Intercept	0.003	0.050	
Cluster: Sites	Intercept	0.025	0.157		Intercept	0.001	0.023	
Sites	Intercept	0.005	0.069		Intercept	0.001	0.023	
	**CWM of abiotically dispersed species**				**CWM of maximum height**			
Intercept	0.509	0.048	10.55	<.001	24.276	0.507	47.90	<.001
Age (19 – 16)	−0.008	0.020	−0.38	.705	−0.205	0.401	−0.50	.617
Age (22 – 16)	−0.013	0.020	−0.65	.517	2.638	0.408	6.46	<.001
Age (OG1 – 16)	−0.247	0.125	−2.06	.039	6.193	1.702	3.64	<.001
Age (OG2 – 16)	−0.258	0.125	−1.97	.048	5.567	1.702	3.27	.001
**Random effects**	**Name**	**Variance**	**SD**		**Name**	**Variance**	**SD**	
Plot: Cluster: Sites	Intercept	0.025	0.158		Intercept	3.214	1.793	
Cluster: Sites	Intercept	0.007	0.084		Intercept	2.676	1.636	
Sites	Intercept	0.005	0.071		Intercept	0.000	0.000	
	**CWM biotically dispersed species**				**Functional richness**			
Intercept	0.462	0.038	12.14	<.001	0.379	0.029	13.27	<.001
Age (19 – 16)	0.014	0.021	0.67	.504	0.038	0.021	1.85	.064
Age (22 – 16)	0.078	0.021	3.71	<.001	0.136	0.021	6.60	<.001
Age (OG1 – 16)	0.329	0.117	2.80	.005	0.329	0.086	3.83	<.001
Age (OG2 – 16)	0.371	0.117	3.16	.002	0.350	0.086	4.08	<.001
**Random effects**	**Name**	**Variance**	**SD**		**Name**	**Variance**	**SD**	
Plot: Cluster: Sites	Intercept	0.025	0.158		Intercept	0.009	0.095	
Cluster: Sites	Intercept	0.011	0.105		Intercept	0.004	0.064	
Sites	Intercept	0.001	0.033		Intercept	0.001	0.030	
	**CWM of small‐fruited species**				**Functional evenness**			
Intercept	0.511	0.055	9.35	<.001	0.693	0.016	44.22	<.001
Age (19 – 16)	−0.005	0.020	−0.24	.810	0.054	0.014	3.79	<.001
Age (22 – 16)	−0.001	0.020	−0.05	.961	0.051	0.014	3.60	<.001
Age (OG1 – 16)	−0.305	0.135	−2.27	.024	0.150	0.053	2.83	.005
Age (OG2 – 16)	−0.319	0.135	−2.37	.018	0.164	0.053	3.10	.002
**Random effects**	**Name**	**Variance**	**SD**		**Name**	**Variance**	**SD**	
Plot: Cluster: Sites	Intercept	0.022	0.147		Intercept	0.005	0.068	
Cluster: Sites	Intercept	0.012	0.109		Intercept	0.001	0.028	
Sites	Intercept	0.007	0.084		Intercept	~0	0.011	
	**CWM of medium‐fruited species**				**Functional divergence**			
Intercept	0.085	0.032	2.67	.008	0.775	0.018	42.12	<.001
Age (19 – 16)	0.005	0.007	0.65	.518	0.007	0.015	0.46	.649
Age (22 – 16)	−0.009	0.007	−1.23	.218	−0.003	0.015	−0.20	.846
Age (OG1 – 16)	0.171	0.073	2.35	.019	−0.068	0.059	−1.16	.248
Age (OG2 – 16)	0.152	0.073	2.10	.036	−0.035	0.059	−0.59	.553
**Random effects**	**Name**	**Variance**	**SD**		**Name**	**Variance**	**SD**	
Plot: Cluster: Sites	Intercept	0.009	0.097		Intercept	0.005	0.068	
Cluster: Sites	Intercept	~0	0.009		Intercept	0.002	0.047	
Sites	Intercept	0.003	0.057		Intercept	0.0002	0.013	
	**CWM of large‐fruited species**				**Functional dispersion**			
Intercept	0.397	0.032	12.430	<.001	0.323	0.008	38.68	<.001
Age (19 – 16)	0.0001	0.018	0.01	.996	0.010	0.007	1.44	.149
Age (22 – 16)	0.005	0.018	0.28	.778	0.024	0.007	3.57	<.001
Age (OG1 – 16)	0.057	0.108	0.53	.597	0.089	0.030	2.94	.003
Age (OG2 – 16)	0.092	0.108	0.85	.393	0.103	0.030	3.43	.001
**Random effects**	**Name**	**Variance**	**SD**		**Name**	**Variance**	**SD**	
Plot: Cluster: Sites	Intercept	0.023	0.153		Intercept	0.002	0.048	
Cluster: Sites	Intercept	0.011	0.105		Intercept	~0	0.016	
Sites	Intercept	~0	~0		Intercept	~0	~0	
	**CWM of pioneer species**				**RaoQ's entropy**			
Intercept	0.567	0.044	12.97	<.001	0.120	0.004	29.48	<.001
Age (19 – 16)	−0.002	0.026	−0.06	.953	0.005	0.003	1.54	.125
Age (22 – 16)	−0.094	0.026	−3.63	<.001	0.011	0.003	3.39	<.001
Age (OG1 – 16)	−0.354	0.117	−3.03	.002	0.047	0.015	3.19	.001
Age (OG2 – 16)	−0.349	0.117	−2.99	.003	0.049	0.015	3.34	.001
**Random effects**	**Name**	**Variance**	**SD**		**Name**	**Variance**	**SD**	
Plot: Cluster: Sites	Intercept	0.014	0.119		Intercept	0.001	0.023	
Cluster: Sites	Intercept	0.006	0.080		Intercept	~0	0.008	
Sites	Intercept	0.004	0.061		Intercept	~0	~0	
	**CWM of nonpioneer light demanders**				**Aboveground biomass**			
Intercept	0.298	0. 027	10.86	<.001	123.182	25.691	4.80	<.001
Age (19 – 16)	−0.002	0.016	−0.10	.921	29.810	8.827	3.38	<.001
Age (22 – 16)	0.0003	0.016	0.02	.983	41.362	8.813	4.69	<.001
Age (OG1 – 16)	0.049	0.095	0.53	.600	468.234	63.761	7.34	<.001
Age (OG2 – 16)	0.057	0.095	0.60	.546	499.772	63.761	7.84	<.001
**Random effects**	**Name**	**Variance**	**SD**		**Name**	**Variance**	**SD**	
Plot: Cluster: Sites	Intercept	0.021	0.146		Intercept	5047	71.04	
Cluster: Sites	Intercept	0.007	0.082		Intercept	2884	53.70	
Sites	Intercept	~0	~0		Intercept	1484	38.52	

*Note*: The secondary forest was sampled in 2011, 2014, and 2017 at 16, 19, and 22 years, respectively, while the reference old‐growth forest was sampled in 2011 (OG1) and 2017 (OG2).

**TABLE A3:** Variation of functional composition (community‐weighted mean—CWM), functional diversity, and aboveground biomass of the secondary forest with distance to forests (old‐growth forest or forest fragment) in Kibale National Park, Uganda.

Fixed effects:	Estimate	SE	*t*	*p*	Estimate	SE	*t*	*p*
	**CWM of forest‐dependent species**				**CWM of shade‐tolerant species**			
Intercept	0.883	0.089	9.839	<.001	0.151	0.046	3.269	.001
Distance to forests	−0.101	0.027	−3.700	<.001	−0.013	0.011	−1.157	.247
Age 19	−0.113	0.069	−1.632	.103				
Age 22	0.040	0.069	0.584	.559				
Distance: Age 19	0.074	0.022	3.372	<.001				
Distance: Age 22	0.032	0.022	1.479	.139				
**Random effects**	**Name**	**Variance**	**SD**		**Name**	**Variance**	**SD**	
Plot: Cluster: Sites	Intercept	0.011	0.104		Intercept	0.005	0.073	
Cluster: Sites	Intercept	0.015	0.123		Intercept	0.002	0.044	
Sites	Intercept	0.003	0.057		Intercept	0.004	0.064	
	**CWM of forest nondependent species**				**CWM of wood density**			
Intercept	0.257	0.095	2.711	.007	0.623	0.022	28.401	<.001
Distance to forests	0.019	0.028	0.702	.483	−0.008	0.006	−1.195	.232
Age 19	−0.014	0.049	−0.277	.782				
Age 22	−0.111	0.049	−2.255	.024				
Distance: Age 19	−0.002	0.016	−0.129	.897				
Distance: Age 22	0.034	0.016	2.172	.029				
**Random effects**	**Name**	**Variance**	**SD**		**Name**	**Variance**	**SD**	
Plot: Cluster: Sites	Intercept	0.026	0.161		Intercept	0.002	0.050	
Cluster: Sites	Intercept	0.017	0.130		Intercept	0.001	0.024	
Sites	Intercept	0.005	0.069		Intercept	0.0004	0.019	
	**CWM of abiotically dispersed species**				**CWM of Maximum height**			
Intercept	0.468	0.079	5.957	<.001	0.623	0.022	28.401	<.001
Distance to forests	0.012	0.022	0.546	.585	0.008	0.006	−1.195	.352
**Random effects**	**Name**	**Variance**	**SD**		**Name**	**Variance**	**SD**	
Plot: Cluster: Sites	Intercept	0.026	0.163		Intercept	0.022	0.148	
Cluster: Sites	Intercept	0.008	0.090		Intercept	0.005	0.074	
Sites	Intercept	0.005	0.074		Intercept	~0	~0	
	**CWM of biotically dispersed species**				**Functional richness**			
Intercept	0.533	0.078	6.844	<.001	0.448	0.052	8.619	<.001
Distance to forests	−0.025	0.024	−1.039	.299	−0.003	0.016	−0.216	.829
Age (19 – 16)	0.029	0.049	0.590	.555				
Age (22 – 16)	0.173	0.049	3.556	<.001				
Distance: Age 19	−0.005	0.015	−0.333	.739				
Distance: Age 22	−0.033	0.015	−2.149	.032				
**Random effects**	**Name**	**Variance**	**SD**		**Name**	**Variance**	**SD**	
Plot: Cluster: Sites	Intercept	0.026	0.162		Intercept	0.008	0.073	
Cluster: Sites	Intercept	0.009	0.099		Intercept	0.005	0.073	
Sites	Intercept	0.001	0.028		Intercept	0.001	0.025	
	**CWM of small‐fruited species**				**Functional evenness**			
Intercept	0.455	0.088	5.184	<.001	0.706	0.029	24.738	<.001
Distance to forests	0.021	0.025	0.832	.405	0.011	0.009	1.212	.226
**Random effects**	**Name**	**Variance**	**SD**		**Name**	**Variance**	**SD**	
Plot: Cluster: Sites	Intercept	0.021	0.146		Intercept	0.005	0.074	
Cluster: Sites	Intercept	0.014	0.119		Intercept	0.001	0.029	
Sites	Intercept	0.007	0.083		Intercept	~0	~0	
	**CWM of medium‐fruited species**				**Functional divergence**			
Intercept	0.098	0.043	2.300	.021	0.857	0.027	32.166	<.001
Distance to forests	−0.005	0.009	−0.559	.576	−0.029	0.008	−3.448	.001
**Random effects**	**Name**	**Variance**	**SD**		**Name**	**Variance**	**SD**	
Plot: Cluster: Sites	Intercept	0.008	0.092		Intercept	0.005	0.069	
Cluster: Sites	Intercept	~0	~0		Intercept	0.001	0.067	
Sites	0.004	0.063			Intercept	~0	~0	
	**CWM of large‐fruited species**				**Functional dispersion**			
Intercept	0.449	0.068	6.593	<.001	0.346	0.019	18.594	<.001
Distance to forests	−0.018	0.021	−0.834	.404	−0.004	0.006	−0.684	.494
**Random effects**	**Name**	**Variance**	**SD**		**Name**	**Variance**	**SD**	
Plot: Cluster: Sites	Intercept	0.024	0.154		Intercept	0.002	0.049	
Cluster: Sites	Intercept	0.009	0.099		Intercept	0.0004	0.020	
Sites	Intercept	~0	~0		Intercept	~0	~0	
	**CWM of pioneer species**				**RaoQ's entropy**			
Intercept	0.392	0.053	7.423	<.001	0.131	0.009	14.413	<.001
Distance to forests	0.052	0.016	3.276	.001	−0.002	0.003	−0.686	.493
**Random effects**	**Name**	**Variance**	**SD**		**Name**	**Variance**	**SD**	
Plot: Cluster: Sites	Intercept	0.014	0.118		Intercept	0.001	0.024	
Cluster: Sites	Intercept	0.002	0.045		Intercept	0.0001	0.010	
Sites	Intercept	0.001	0.038		Intercept	~0	~0	
	**CWM of nonpioneer light demanders**				**Aboveground biomass**			
Intercept	0.399	0.057	6.988	<.001	170.100	36.158	4.704	<.001
Distance to forests	−0.035	0.018	−1.963	.049	−9.013	10.941	−0.824	.410
**Random effects**	**Name**	**Variance**	**SD**		**Name**	**Variance**	**SD**	
Plot: Cluster: Sites	Intercept	0.022	0.148		Intercept	4977	70.55	
Cluster: Sites	Intercept	0.005	0.006		Intercept	2668	51.65	
Sites	Intercept	~0	~0		Intercept	506	22.49	
