# Supplementary material for: Tree functional composition, functional diversity, and aboveground biomass show dissimilar trajectories in a tropical secondary forest restored through assisted natural regeneration
Source: Ecol Evol. 2023 Mar 12;13(3):e9870. doi: 10.1002/ece3.9870 (PMC10008352; doi:10.1002/ece3.9870)
Supplement: Supplementary file 1 — Table S1 [file ECE3-13-e9870-s001.docx]

**SUPPORTING INFORMATION**

**TABLE S1** Abundance and functional traits of tree species recorded in the secondary forest (SF) and old-growth forest (OGF) in Kibale National Park, Uganda. In habitat type, openhab = open habitat, fordep = forest-dependent, fornondep = forest nondependent, in successional group, PION = pioneer, NPLD = Non-pioneer light demander, SHTO = Shade-tolerant, SASP = savannah species, SWSP = Swamp species

| **Species** | Family | Habitat type | Fruit size | Fruit size category | Successional group | Dispersal | Maximum Height (m) | Wood density (g/cm^3) | **OGF** | **SF** | **Total** |
| --- | --- | --- | --- | --- | --- | --- | --- | --- | --- | --- | --- |
| *Acacia abyssinica* | Mimosoideae | openhab | >5cm | Large | SASP | Abiotic | 16.3 | 0.62 |  | 4 | 4 |
| *Albizia adianthifolia* | Mimosoideae | fornondep | >5cm | Large | PION | Abiotic | 19.9 | 0.55 |  | 2 | 2 |
| *Albizia coriaria* | Mimosoideae | fornondep | >5cm | Large | SASP | Abiotic | 21.4 | 0.65 |  | 2 | 2 |
| *Albizia grandibracteata* | Mimosoideae | fornondep | >5cm | Large | NPLD | Abiotic | 25.5 | 0.48 |  | 1 | 1 |
| *Albizia gummifera* | Mimosoideae | openhab | >5cm | Large | NPLD | Abiotic | 18.7 | 0.67 |  | 1 | 1 |
| *Albizia versicolor* | Mimosoideae | openhab | >5cm | Large | NPLD | Abiotic | 14.3 | 0.67 |  | 1 | 1 |
| *Albizia zygia* | Mimosoideae | fornondep | >5cm | Large | NPLD | Abiotic | 28.8 | 0.67 | 4 | 142 | 146 |
| *Allophylus abyssinicus* | Sapindaceae | fordep | <1cm | Small | NPLD | Biotic | 14.0 | 0.61 |  | 1 | 1 |
| *Allophylus africanus* | Sapindaceae | openhab | <1cm | Small | NPLD | Biotic | 14.5 | 0.54 |  | 6 | 6 |
| *Aningeria altissima* | Sapotaceae | fordep | 1-3cm | Medium | NPLD | Biotic | 41.8 | 0.58 | 3 |  | 3 |
| *Antidesma laciniatum* | Euphorbiaceae | fordep | <1cm | Small | SHTO | Biotic | 15.9 | 0.59 |  | 28 | 28 |
| *Aphania senegalensis* | Sapindaceae | fornondep | 1-3cm | Medium | SHTO | Biotic | 14.7 | 0.752 |  | 8 | 8 |
| *Balanites wilsoniana* | Balanitaceae | fordep | >5cm | Large | NPLD | Biotic | 34.3 | 0.58 |  | 3 | 3 |
| *Bequaertiodendron natalense* | Sapotaceae | fordep | 1-3cm | Medium | SHTO | Biotic | 38.2 | 0.7 | 2 |  | 2 |
| *Bersama abyssinica* | Melianthaceae | fornondep | 1-3cm | Medium | PION | Biotic | 22.6 | 0.62 |  | 3 | 3 |
| *Blighia unijugata* | Sapindaceae | fordep | <1cm | Small | SHTO | Biotic | 23.0 | 0.61 |  | 22 | 22 |
| *Blighia welwitschii* | Sapindaceae | fordep | 3.1-5cm | Large | NPLD | Biotic | 27.4 | 0.86 | 1 |  | 1 |
| *Boscia angustifolia* | Capparaceae | fordep | <1cm | Small | SASP | Biotic | 28.3 | 0.59 | 2 |  | 2 |
| *Bridelia micrantha* | Euphorbiaceae | fornondep | <1cm | Small | PION | Biotic | 23.0 | 0.58 |  | 195 | 195 |
| *Bridelia ndellensis* | Euphorbiaceae | fornondep | <1cm | Small | PION | Biotic | 14.6 | 0.687 |  | 1 | 1 |
| *Caloncoba crepiniana* | Flacourtiaceae | fornondep | >5cm | Large | PION | Biotic | 13.5 | 0.72 |  | 2 | 2 |

**TABLE S1 Continued**

| *Species* | Family | Habitat type | Fruit size | Fruit size category | Successional group | Dispersal | Maximum Height (m) | Wood density (g/cm^3) | OGF | SF | Total |
| --- | --- | --- | --- | --- | --- | --- | --- | --- | --- | --- | --- |
| *Caloncoba schwernsferthii* | Flacourtiaceae | fornondep | >5cm | Large | PION | Biotic | 24.6 | 0.72 |  | 17 | 17 |
| *Cassia spectabilis* | Caesalpinioideae | fornondep | >5cm | Large | PION | Abiotic | 29.0 | 0.48 |  | 144 | 144 |
| *Cassipourea congensis* | Rhizophoraceae | fordep | <1cm | Small | SHTO | Biotic | 6.8 | 0.66 |  | 1 | 1 |
| *Cassipourea gummiflua* | Rhizophoraceae | fornondep | <1cm | Small | NPLD | Biotic | 10.5 | 0.54 | 1 |  | 1 |
| *Celtis africana* | Ulmaceae | fordep | <1cm | Small | PION | Biotic | 36.8 | 0.73 | 11 | 94 | 105 |
| *Celtis durandii* | Ulmaceae | fordep | <1cm | Small | PION | Biotic | 39.3 | 0.54 | 4 | 179 | 183 |
| *Celtis mildbraedii* | Ulmaceae | fordep | <1cm | Small | SHTO | Biotic | 40.3 | 0.69 | 1 | 8 | 9 |
| *Celtis zenkeri* | Ulmaceae | fordep | <1cm | Small | NPLD | Biotic | 36.7 | 0.73 |  | 22 | 22 |
| *Chaetacme aristata* | Ulmaceae | fordep | 1-3cm | Medium | PION | Biotic | 17.6 | 0.56 | 2 | 6 | 8 |
| *Chrysophyllum albidum* | Sapotaceae | fordep | 3.1-5cm | Large | SHTO | Biotic | 36.2 | 0.69 | 19 | 23 | 42 |
| *Chrysophyllum gorungosanum* | Sapotaceae | fordep | 3.1-5cm | Large | SHTO | Biotic | 29.6 | 0.63 | 2 |  | 2 |
| *Clausena anisata* | Rutaceae | fornondep | <1cm | Small | PION | Biotic | 6.2 | 0.57 | 1 |  | 1 |
| *Coffea canephora* | Rubiaceae | fordep | 1-3cm | Medium | SHTO | Biotic | 10.1 | 0.63 |  | 1 | 1 |
| *Coffea liberica* | Rubiaceae | fordep | 1-3cm | Medium | SHTO | Biotic | 11.5 | 0.63 |  | 5 | 5 |
| *Cola bracteata* | Sterculiaceae | fordep | >5cm | Large | NPLD | Abiotic | 17.3 | 0.63 | 1 |  | 1 |
| *Cordia millenii* | Boraginaceae | fordep | 3.1-5cm | Large | PION | Biotic | 31.2 | 0.41 | 1 |  | 1 |
| *Crassocephalum mannii* | Sapindaceae | fornondep | 1-3cm | Medium | SHTO | Abiotic | 18.5 | 0.331 | 1 |  | 1 |
| *Crossonephelum africanus* | Sapindaceae | fornondep | 1-3cm | Medium | SHTO | Biotic | 16.5 | 0.67 | 1 | 1 | 2 |
| *Croton macrostachyus* | Euphorbiaceae | fordep | <1cm | Small | NPLD | Biotic | 36.8 | 0.61 | 1 | 14 | 15 |
| *Croton megalocarpus* | Euphorbiaceae | fordep | 1-3cm | Medium | NPLD | Abiotic | 33.6 | 0.73 | 2 |  | 2 |
| *Dialium excelsum* | Caesalpinioideae | fordep | 1-3cm | Medium | PION | Abiotic | 13.7 | 0.96 | 3 |  | 3 |
| *Diospyros abyssinica* | Ebenaceae | fordep | <1cm | Small | PION | Biotic | 9.6 | 0.78 | 3 | 55 | 58 |
| *Diospyros mespiliformis* | Ebenaceae | fordep | 1-3cm | Medium | PION | Biotic | 31.1 | 0.98 | 8 | 83 | 91 |
| *Dissotis perkinsiae* | Melastomataceae | fornondep | >5cm | Large | SHTO | Abiotic | 9.9 | 0.7 | 6 | 2 | 8 |
| *Dombeya mukole* | Sterculiaceae | fordep | 1-3cm | Medium | NPLD | Abiotic | 27.3 | 0.48 |  | 17 | 17 |
| *Entandrophragma angolense* | Meliaceae | fordep | >5cm | Large | NPLD | Abiotic | 7.0 | 0.63 | 1 |  | 1 |
| *Erythrina abyssinica* | Papilionaceae | fornondep | >5cm | Large | PION | Abiotic | 17.8 | 0.38 |  | 26 | 26 |

**TABLE S1 Continued**

| *Species* | Family | Habitat type | Fruit size | Fruit size category | Successional group | Dispersal | Maximum Height (m) | Wood density (g/cm^3) | OGF | SF | Total |
| --- | --- | --- | --- | --- | --- | --- | --- | --- | --- | --- | --- |
| *Erythrina excelsa* | Papilionideae | fordep | >5cm | Large | PION | Abiotic | 16.9 | 0.64 |  | 1 | 1 |
| *Euadenia eminens* | Capparidaceae | fordep | >5cm | Large | SHTO | Abiotic | 16.3 | 0.42 |  | 58 | 58 |
| *Eucalyptus saligna* | Myrtaceae | undet | <1cm | Small | NPLD | Abiotic | 41.5 | 0.83 |  | 9 | 9 |
| *Euphorbia sp* | Euphorbiaceae | openhab | <1cm | Small | Unknown | Biotic | 12.1 | 0.47 |  | 2 | 2 |
| *Ficus exasperata* | Moraceae | fornondep | 1-3cm | Medium | PION | Biotic | 23.9 | 0.34 |  | 1 | 1 |
| *Ficus mucuso* | Moraceae | fordep | 1-3cm | Medium | PION | Biotic | 32.1 | 0.48 |  | 10 | 10 |
| *Ficus natalensis* | Moraceae | fornondep | <1cm | Small | PION | Biotic | 17.1 | 0.44 |  | 1 | 1 |
| *Ficus ovata* | Moraceae | fornondep | <1cm | Small | PION | Biotic | 14.0 | 0.48 |  | 1 | 1 |
| *Ficus sur* | Moraceae | fornondep | 1-3cm | Medium | PION | Biotic | 27.5 | 0.48 |  | 3 | 3 |
| *Ficus vallis-choudae* | Moraceae | fordep | <1cm | Small | PION | Biotic | 16.9 | 0.441 |  | 3 | 3 |
| *Ficus variifolia* | Moraceae | fordep | <1cm | Small | PION | Biotic | 28.0 | 0.4 | 1 |  | 1 |
| *Flueggea virosa* | Euphorbiaceae | fornondep | <1cm | Small | NPLD | Biotic | 12.2 | 0.77 |  | 28 | 28 |
| *Funtumia africana* | Apocynaceae | fordep | >5cm | Large | NPLD | Abiotic | 37.1 | 0.48 | 10 | 298 | 308 |
| *Funtumia elastica* | Apocynaceae | fordep | >5cm | Large | NPLD | Abiotic | 38.2 | 0.475 | 22 | 5 | 27 |
| *Harrisonia abyssinica* | Simaroubaceae | fordep | <1cm | Small | PION | Biotic | 15.6 | 0.785 |  | 22 | 22 |
| *Holoptelea grandis* | Ulmaceae | fordep | <1cm | Small | PION | Abiotic | 36.0 | 0.74 | 1 |  | 1 |
| *Jacaranda mimosifolia* | Bignoniaceae | openhab | 3.1-5cm | Large | PION | Abiotic | 18.6 | 0.55 |  | 3 | 3 |
| *Jatropha curcus* | Euphorbiaceae | openhab | 3.1-5cm | Large | NPLD | Biotic | 13.0 | 0.3 |  | 2 | 2 |
| *Kigelia africana* | Bignoniaceae | fornondep | >5cm | Large | NPLD | Biotic | 8.4 | 0.61 |  | 23 | 23 |
| *Linociera nilotica* | Oleaceae | fordep | 1-3cm | Medium | NPLD | Biotic | 15.9 | 0.48 |  | 1 | 1 |
| *Maesa lanceolata* | Myrsinaceae | fornondep | <1cm | Small | NPLD | Biotic | 12.4 | 0.68 |  | 5 | 5 |
| *Maesopsis eminii* | Rhamnaceae | fordep | 1-3cm | Medium | PION | Biotic | 25.5 | 0.46 |  | 4 | 4 |
| *Mangifera indica* | Anacardiaceae | fornondep | >5cm | Large | PION | Biotic | 2.1 | 0.62 |  | 3 | 3 |
| *Markhamia platycalyx* | Bignoniaceae | fordep | >5cm | Large | PION | Abiotic | 33.1 | 0.54 | 5 | 15 | 20 |
| *Maytenus ovata* | Celastraceae | fordep | <1cm | Small | NPLD | Biotic | 6.6 | 0.48 |  | 1 | 1 |
| *Maytenus serratus* | Celastraceae | openhab | <1cm | Small | PION | Biotic | 7.0 | 0.66 |  | 1 | 1 |
| *Maytenus undata* | Celastraceae | fordep | <1cm | Small | NPLD | Biotic | 24.8 | 0.73 | 3 | 22 | 25 |

**TABLE S1 Continued**

| Species | Family | Habitat type | Fruit size | Fruit size category | Successional group | Dispersal | Maximum Height (m) | Wood density (g/cm^3) | OGF | SF | Total |
| --- | --- | --- | --- | --- | --- | --- | --- | --- | --- | --- | --- |
| *Milicia excelsa* | Moraceae | fordep | 3.1-5cm | Large | PION | Biotic | 14.9 | 0.65 | 1 |  | 1 |
| *Millettia dura* | Papilionaceae | fordep | >5cm | Large | NPLD | Abiotic | 17.0 | 0.7 |  | 29 | 29 |
| *Mimulopsis arborescens* | Acanthaceae | fornondep | 3.1-5cm | Large | PION | Abiotic | 21.0 | 0.79 | 1 |  | 1 |
| *Mimusops bagshawei* | Sapotaceae | fordep | 1-3cm | Medium | NPLD | Biotic | 35.8 | 0.77 | 5 |  | 5 |
| *Mimusops kummel* | Annonaceae | fornondep | >5cm | Large | SHTO | Biotic | 31.2 | 0.64 | 30 |  | 30 |
| *Monodora angolensis* | Annonaceae | fordep | >5cm | Large | SHTO | Biotic | 45.8 | 0.54 | 2 |  | 2 |
| *Monodora myristica* | Annonaceae | fordep | >5cm | Large | SHTO | Biotic | 39.9 | 0.49 | 5 | 2 | 7 |
| *Morus alba* | Moraceae | openhab | 1-3cm | Medium | PION | Biotic | 37.3 | 0.62 | 4 | 1 | 5 |
| *Neoboutonia macrocalyx* | Euphorbiaceae | fordep | <1cm | Small | PION | Abiotic | 38.3 | 0.39 | 1 |  | 1 |
| *Olea welwitschii* | Oleaceae | fordep | <1cm | Small | NPLD | Biotic | 39.2 | 0.77 | 9 | 9 | 18 |
| *Oncoba spinosa* | Flacourtiaceae | fornondep | >5cm | Large | SASP | Abiotic | 9.8 | 0.58 |  | 1 | 1 |
| *Pancovia sp nr turbinata* | Sapindaceae | fordep | 1-3cm | Medium | SHTO | Biotic | 25.6 | 0.78 | 1 |  | 1 |
| *Parinari excelsa* | Chrysobalanaceae | fordep | 3.1-5cm | Large | SHTO | Biotic | 16.9 | 0.87 |  | 1 | 1 |
| *Phoenix reclinata* | Palmae | fornondep | 1-3cm | Medium | NPLD | Biotic | 5.1 | 0.74 |  | 1 | 1 |
| *Pleiocarpa pycnantha* | Apocynaceae | fordep | 1-3cm | Medium | SWSP | Biotic | 9.1 | 0.64 | 2 |  | 2 |
| *Podocarpus gracilior* | Podocarpaceae | fordep | 1-3cm | Medium | SHTO | Biotic | 27.3 | 0.53 |  | 1 | 1 |
| *Polyscias fulva* | Araliaceae | fornondep | <1cm | Small | PION | Biotic | 26.1 | 0.38 | 2 | 1 | 3 |
| *Prunus africana* | Rosaceae | fornondep | <1cm | Small | NPLD | Biotic | 34.0 | 0.55 | 5 | 4 | 9 |
| *Pseudospondias microcarpa* | Anacardiaceae | fornondep | 1-3cm | Medium | SWSP | Biotic | 16.5 | 0.62 |  | 3 | 3 |
| *Psorospernum febrifugum* | Guttiferae | openhab | <1cm | Small | NPLD | Biotic | 5.6 | 0.69 | 1 |  | 1 |
| *Pterygota mildbraedii* | Sterculiaceae | fordep | 1-3cm | Medium | NPLD | Abiotic | 39.1 | 0.59 | 2 |  | 2 |
| *Rauvolfia caffra* | Apocynaceae | fordep | <1cm | Small | PION | Biotic | 10.9 | 0.47 |  | 1 | 1 |
| *Rauvolfia oxyphylla* | Apocynaceae | fordep | <1cm | Small | PION | Biotic | 17.6 | 0.47 |  | 1 | 1 |
| *Rauvolfia vomitoria* | Apocynaceae | fordep | <1cm | Small | PION | Biotic | 22.4 | 0.47 |  | 38 | 38 |
| *Rothmannia longiflora* | Rubiaceae | fornondep | >5cm | Large | SHTO | Biotic | 13.3 | 0.64 |  | 10 | 10 |
| *Rothmannia urcelliformis* | Rubiaceae | fordep | >5cm | Large | SHTO | Biotic | 7.7 | 0.54 |  | 7 | 7 |
| *Rothmannia whitfieldii* | Rubiaceae | fordep | >5cm | Large | SHTO | Biotic | 10.8 | 0.64 |  | 1 | 1 |

**TABLE S1 Continued**

| *Species* | Family | Habitat type | Fruit size | Fruit size category | Successional group | Dispersal | Maximum Height (m) | Wood density (g/cm^3) | OGF | SF | Total |
| --- | --- | --- | --- | --- | --- | --- | --- | --- | --- | --- | --- |
| *Sapium leonardii-crispi* | Euphorbiaceae | fordep | <1cm | Small | PION | Abiotic | 17.8 | 0.46 |  | 4 | 4 |
| *Shirakiopsis elliptica* | Euphorbiaceae | fordep | <1cm | Small | PION | Abiotic | 26.2 | 0.61 |  | 344 | 344 |
| *Sorindeia submontana* | Anacardiaceae | fordep | 1-3cm | Medium | SHTO | Biotic | 14.3 | 0.59 |  | 1 | 1 |
| *Spathodea campanulata* | Bignoniaceae | fordep | >5cm | Large | PION | Abiotic | 28.5 | 0.23 |  | 72 | 72 |
| *Strombosia scheffleri* | Olacaceae | fordep | 1-3cm | Medium | SHTO | Biotic | 32.9 | 0.86 | 8 |  | 8 |
| *Strombosiopsis tetrandra* | Olacaceae | fordep | 1-3cm | Medium | SHTO | Biotic | 8.7 | 0.86 |  | 1 | 1 |
| *Tabebuia rosea* | Bignoniaceae | openhab | >5cm | Large | SASP | Abiotic | 21.0 | 0.53 |  | 1 | 1 |
| *Tabernaemontana holstii* | Apocynaceae | fordep | >5cm | Large | SHTO | Biotic | 14.6 | 0.54 | 5 | 14 | 19 |
| *Tabernaemontana johnstonii* | Apocynaceae | fordep | >5cm | Large | SHTO | Biotic | 7.6 | 0.54 | 1 | 1 | 2 |
| *Teclea nobilis* | Rutaceae | fornondep | <1cm | Small | SHTO | Biotic | 29.3 | 0.75 | 3 | 4 | 7 |
| *Thevetia peruviana* | Apocynaceae | fornondep | 3.1-5cm | Large | SHTO | Biotic | 9.1 | 0.72 |  | 27 | 27 |
| *Treculia africana* | Moraceae | fordep | >5cm | Large | NPLD | Biotic | 22.7 | 0.54 | 2 | 3 | 5 |
| *Trichilia africana* | Meliaceae | fordep | 1-3cm | Medium | NPLD | Biotic | 35.1 | 0.51 | 10 | 7 | 17 |
| *Trichilia dregeana* | Meliaceae | fordep | 1-3cm | Medium | NPLD | Biotic | 18.7 | 0.48 |  | 8 | 8 |
| *Trichilia prieureana* | Meliaceae | fornondep | 1-3cm | Medium | NPLD | Biotic | 15.9 | 0.75 |  | 1 | 1 |
| *Trilepisium madagascariensis* | Moraceae | fordep | 1-3cm | Medium | NPLD | Biotic | 50.1 | 0.46 | 2 |  | 2 |
| *Uvariopsis congensis* | Annonaceae | fordep | <1cm | Small | SHTO | Biotic | 24.0 | 0.66 | 14 | 37 | 51 |
| *Voacanga thouarsii* | Apocynaceae | openhab | <1cm | Small | SWSP | Biotic | 17.5 | 0.7 |  | 1 | 1 |
| *Warburgia ugandensis* | Canellaceae | fordep | >5cm | Large | NPLD | Biotic | 29.9 | 0.86 |  | 26 | 26 |
